# A picture is worth a thousand data points: an imagery dataset of paired shrub-open microsites within the Carrizo Plain National Monument

**DOI:** 10.1186/s13742-016-0145-2

**Published:** 2016-09-27

**Authors:** Taylor J. Noble, Christopher J. Lortie, Michael Westphal, H. Scott Butterfield

**Affiliations:** 1 Department of Biology, York University, 244 Farquharson Life Sciences Building, 4700 Keele Street, M3J 1P3 Toronto, ON Canada; 2 U.S. Bureau of Land Management, Central Coast Field Office, Marina, CA USA; 3 The Nature Conservancy, San Francisco, CA USA

**Keywords:** Blunt-nosed leopard lizard, Camera trapping, Carrizo plain national monument, Ephedra californica, Facilitation, San Joaquin desert, San Joaquin kit fox, San Joaquin valley, San Luis Obispo county

## Abstract

**Background:**

Carrizo Plain National Monument (San Joaquin Desert, California, USA) is home to many threatened and endangered species including the blunt-nosed leopard lizard (*Gambelia sila*). Vegetation is dominated by annual grasses, and shrubs such as Mormon tea (*Ephedra californica*), which is of relevance to our target species, the federally listed blunt-nosed leopard lizard, and likely also provides key ecosystem services. We used relatively nonintrusive camera traps, or trail cameras, to capture interactions between animals and these shrubs using a paired shrub-open deployment. Cameras were placed within the shrub understory and in open microhabitats at ground level to estimate animal activity and determine species presence.

**Findings:**

Twenty cameras were deployed from April 1st, 2015 to July 5th, 2015 at paired shrub-open microsites at three locations. Over 425,000 pictures were taken during this time, of which 0.4 % detected mammals, birds, insects, and reptiles including the blunt-nosed leopard lizard. Trigger rate was very high on the medium sensitivity camera setting in this desert ecosystem, and rates did not differ between microsites.

**Conclusions:**

Camera traps are an effective, less invasive survey method for collecting data on the presence or absence of desert animals in shrub and open microhabitats. A more extensive array of cameras within an arid region would thus be an effective tool to estimate the presence of desert animals and potentially detect habitat use patterns.

## Background

Camera traps, or trail cameras, automatically take pictures of animals when remotely deployed in the field [1–3]. Most camera traps available today have a passive infrared sensor [1, 2], which detects changes in heat-in-motion, allowing moving animals to be detected against background temperature and motion [2]. Camera traps are often used to determine the presence or absence of a species in an area [1, 2]. They can also be used to calculate density, determine relative abundance, and explore habitat occupancy of animal species [1, 3, 4].

Using cameras means that animals do not need to be handled and overall disturbance to the study area is reduced [5]. However, an observer effect still exists. Many animals can sense cameras from the sounds they make as they are triggered, or from the camera flash, but, in most cases, this will not prevent the animal from being detected by the cameras [6]. Unlike transects or other visual surveys of animal species, cameras can be used to maintain a continuous survey of an area, which makes them effective at detecting rare or elusive species [5, 7]. Pictures from camera traps also offer a digital record of an observation, which can be an advantage over a single observer. The imagery produced from camera traps also has important uses in advocacy and engagement through citizen science, since it often shows rare or elusive species, which can be useful in raising awareness of and interest in the conservation and protection of these species [1].

However, reporting and use of camera traps is not always transparent [3]. There are many different camera types and brands available today. Specifications for each model and camera settings such as trigger rate, trigger delay, and number of images recorded per trigger may not be provided in the methods. Images produced from camera trap surveys are often not available [3]. By improving camera-trapping practices and sharing primary data in the form of imagery we have an opportunity to discover novel quantitative patterns that would not be visible with individual camera trap studies.

Camera traps have been used in many different types of ecosystems including semi-arid grasslands such as the Serengeti [8], mountainous regions such as the Himalayas [9], and tropical forests such as the Amazon rainforest [10]. Camera traps are also commonly used in desert systems such as the deserts of California, USA [11–13].

Californian deserts are significant ecosystems that are not only subject to climate perturbations [14, 15], but are also home to many species of conservation concern [16]. Though often classified as semi-arid grassland, it has been recognized that the southern San Joaquin Valley of California has many desert characteristics such as low rainfall and desert-tolerant species. Therefore, some researchers classify it as a unique desert called the San Joaquin Desert [16]. Desert habitat within this region remains at less than 5 % of its historical distribution [16, 17]. This region has largely been converted to irrigated agriculture and urban land uses [16, 17]. It hosts a suite of endangered, threatened, and sensitive species including the endangered San Joaquin kit fox (*Vulpes macrotis mutica*), giant kangaroo rat (*Dipodomys ingens*), and blunt-nosed leopard lizard (*Gambelia sila*) [16, 18, 19]. Consequently, surveying animals in these remnant habitats within the region is important for conservation and management.

Three core remnants of the San Joaquin Desert ecosystem remain in California [17]. Carrizo Plain National Monument (35.1914° N, 119.7929° W), located in southeastern San Luis Obispo County, is the largest [16]. Precipitation at the monument ranges from 15 cm in the southeast to 25 cm in the northwest [20]. The present study took place on the Elkhorn Plain within the Monument, a plain that was elevated and separated from the main valley floor by tectonic activity of the San Andreas Fault [21].

The area has been heavily invaded by non-native annual grasses including *Bromus madritensis*, *Erodium cicutarium*, and *Hordeum murinum* [22, 23]. The dominant shrubs are Mormon tea (*Ephedra californica*) and saltbush (*Atriplex polycarpa*) [24]. In general, shrubs are an important feature in the landscape for other species of plants [25, 26] as well as animals [27]. These foundation plant species typically facilitate other species by providing shelter, refuge, and resources, such as additional prey or moisture [27, 28]. These positive effects can be particularly important in harsh environments, such as deserts, where resources are often scarce or species are close to the limits of their climatic tolerances [27, 29–31]. The milder microclimate found under a shrub can be beneficial to both plant and animal species [28]. Hence, within the San Joaquin Desert, ephedra are a useful focus for structured plant-animal surveys, and are likely an important consideration for habitat management and monitoring, whether via camera traps or other methods.

A paired shrub-open method of camera trap deployment was used to examine the ecological importance of shrubs compared to other species within this specific desert region. The primary purpose of this study, and these subsequent data, was to capture the presence of a flagship species-the blunt-nosed leopard lizard-within the region. This species is listed, endangered, and often at low population densities [29, 32]. Camera traps are often used as a method of detection for endangered species [8, 10, 11]. The spatial partitioning of deserts into shrub-open habitat classes is a common method used to study positive interactions in deserts [33]. This method is often used to examine shrub-annual plant interactions, but is not commonly applied to the ecological study of shrub-animal interactions. Image data from camera traps are becoming increasingly common as a form of evidence for conservation and wildlife biologists, as well as ecologists [1–4, 8, 34, 35]. This dataset thus provides important evidence for animal presence, potential abundance estimates, and for desert animals, including an endangered species, within a national monument. Analyses of these patterns are beyond the scope of this data contribution, but summary statistics associated with the camera trapping protocol are provided as an indication of the merit of sharing these data.

## Data description

### Camera deployment

**Fig. 1:**
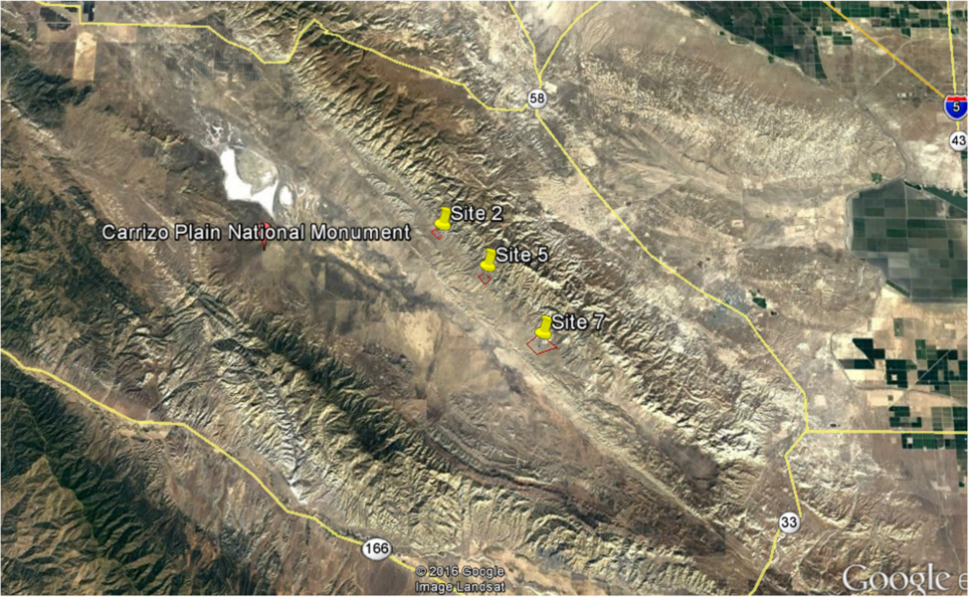
Map of sites with Carrizo Plain National Monument. Sites used for camera deployment were located along Elkhorn Road within Carrizo Plains National Monument

Cameras were set at three sites in the Elkhorn Plain within Carrizo National Monument: 1) 35.197505°,−119.71552° (named site 2); 2) 35.163363°,−119.674° (named site 5); and 3) 35.11432°,−119.6209° (named site 7) (Fig. 1). Sites were at least 2 km apart and at least 1 km^2^. Sites located on public land held by the U.S. Bureau of Land Management were selected based on known presence of the target species (blunt-nosed leopard lizards), and presence of shrubs in the area to allow shrub-open paired microsites for camera deployment. The daily movement of blunt-nosed leopard lizards usually ranges between 65 and 110 m but can reach up to 300 m [36]. Therefore, the 2 km spacing between sites was selected to provide a sufficient buffer for extreme dispersal events of this and other species, to ensure that small mammals and lizard sightings at one site on a given day were independent of sightings at other sites.

A total of 14 Primos camera traps (Primos Hunting, Flora, MS, USA) and 6 Reconyx camera traps (Reconyx, Holmen, WI, USA) were deployed from April 1st through July 5th, 2015. Both models have a camera of at least 3 megapixels (3.1 MP for the Reconyx, 7 MP for the Primos), with at least 2 sensitivity settings (5 for the Reconyx, 2 for the Primos), and have replaceable batteries. Both models use Secure Digital (SD) cards. Both cameras also contain a passive infrared (PIR) motion sensor for detecting heat-in-motion during the day and night [37, 38]. Cameras were deployed in a paired design with 2 cameras focused on microsites located near a shrub; one camera facing the north side of a shrub between 1 and 2 m from the shrub, the other facing away from the shrub focused on an open area between 5 and 10 m away from the north side of the surveyed shrub. This area was at least 5 m away from any other shrub. Cameras were attached to 20 cm pegs anchored firmly in the ground, with the camera placed between 10 and 15 cm off the ground. Vegetation was left intact and disturbance was minimized. From April until mid-May, cameras were set at each shrub-open pair from sunrise to sunset, and then moved to a new shrub-open pair the next day, for a total of 133 shrub-open pairs sampled during this time. From mid-May to July cameras were deployed in a pattern of one, 4-day deployment followed by two, 1-day deployments. During this time 49 shrub-open pairs were sampled for 4 days, and 61 shrub-open pairs were sampled for 1 day. All deployments were at new shrub-open pairs so as to more intensively sample microhabitats at peak animal activity, while still sampling as many shrub-open pairs as possible. In some instances cameras were deployed for slightly shorter or slightly longer periods of time, depending on when they were able be checked. Exact dates and lengths of each deployment for each camera are listed in the metadata.

### Imagery collected

Sites were surveyed on consecutive days, but the order in which they were surveyed was random. At each site, shrubs were also selected at random to survey as many shrubs as possible over the course of the season. Individual shrubs were not surveyed more than once during the study, to avoid repeated measures and to ensure that desert sites were sampled as widely was possible. At each set of paired shrub-open sites, shrub size (length, width, and height), and distance to nearest 3 shrubs was recorded. Annual plant abundance, annual plant species composition, and annual plant density were recorded using a 0.25 m^2^ quadrat at the location of each camera. Camera settings (picture mode, number of pictures per trigger, and sensitivity level) were also recorded. Cameras were set to record a three-picture burst per trigger followed by a 10 s delay at the highest quality picture setting. The default (highest) sensitivity level setting was used. Camera images numbering 425,369 in Joint Photographic Experts Group (JPEG) format were examined to determine the presence of animals over the season. False triggering of the cameras by grass or other vegetation moving in the wind was common, leading to greater battery power consumption and rapid filling of the memory storage cards. However, this did not lessen the effectiveness of the camera, as animal movement still triggered the camera sensors. Battery life and data storage could last more than 1 week, so no survey time was lost to false triggers. We reviewed 100,000 of the images for content before uploading them to *GigaDB* to ensure image quality, presence of animals, date and temperature stamp, and data integrity.

### Metadata

**Fig. 2:**
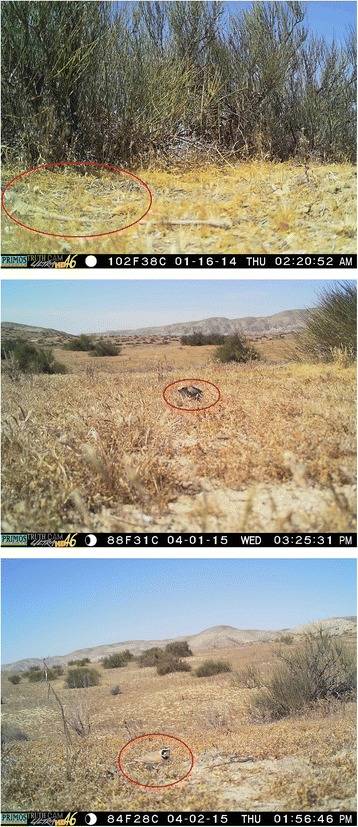
Sample pictures of animals detected by camera traps. Images here show the detection of Image 1: the endangered blunt-nosed leopard lizard (*Gambelia sila*), Image 2: San Joaquin antelope squirrel (*Ammospermophilus nelson*), and Image 3: Horned Lark (*Eremophila alpestris*)

**Fig. 3:**
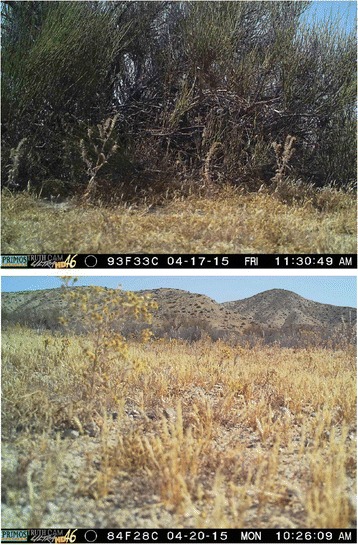
Sample pictures of shrub and open microsites. Cameras were effectively triggered at both shrub (top) and open microsites (bottom) within this desert ecosystem. Microsite type did not have an effect on camera effectiveness and animal images were detected at both shrub and open microsites

Animals were detected in 0.4 % of the reviewed images, including San Joaquin antelope squirrels (*Ammospermophilus nelson*), jackrabbits (*Lepus californicus*), coyotes (*Canis latrans*), blunt-nosed leopard lizards (*Gambelia sila*), whiptail lizards (*Cnemidophorus tigris*), side-blotched lizards (*Uta stansburiana*), loggerhead shrikes (*Lanius ludovicianus*), and grasshopper and butterfly species (Fig. 2). Images from cameras at both shrub and open microsites were clear and readable, and the camera resolution was sufficient under challenging conditions (such as low light) to discern and identify animals during both day and night (Fig. 3). The mean file size for each JPEG image was 750 kb. Filenames were encoded based on the location of the camera, the camera number, and the date the pictures were taken. Metadata provided for each file include the start and end date of the collection, site id, shrub number for that day, whether the camera was at a shrub or open microsite, the direction the camera was facing, the camera number and type, camera settings, and the geographic location. A selection of images containing animals are provided along with the raw imagery data to provide an example of the species detected. Even though the cameras were placed in a desert ecosystem with high ambient temperatures, the cameras did not exceed their maximum operating temperature, and there were no camera failures. It is nonetheless possible that at very high desert temperatures, detection rates might be influenced by background infrared interference during daylight hours [39].

### Potential uses

Camera traps can provide data for a range of basic ecology and wildlife ecology research. A common use of camera traps is to assess the presence or absence of animal species [1, 2]. Cameras are able to survey continuously, which is thus useful in the detection of rare or elusive species [40]. Imaged animals can be identified to the species level, allowing an inventory of species to be created [2]. As with other types of trapping, the method requires an adequate number of days to examine species presence. This can vary by species, but is usually around 30 days [1, 2. 40].

Camera traps are also used to estimate relative species abundance according to the number of times each species triggers the camera-the more triggers by a particular species, the greater is its relative abundance in the area being surveyed [2]. There are, however, limitations to this estimate because an individual animal can trigger multiple images during a short time period or over several days [2, 40]. If specific individuals can be identified from the images, then mark-recapture analysis can be used to obtain a more accurate estimate of abundance [2]. In this type of model, the frequency of marked or known individuals and the frequency of unmarked or new individuals are used to calculate abundance [2, 40]. Though useful, this method is limited because there is potential for misidentification. Not all individuals have identifying marks, or the marks may be hidden, or require subjective classification [40]. Species density may be found using abundance [2, 40]. However, to do this requires calculation of the effective trapping area, or area covered by the cameras [40].

Even if individuals of a species cannot easily be identified, abundance and density may still be calculated using a random encounter model [41]. This method uses the species’ trapping rate, the speed of the captured animal, and the location of the animal relative to the camera when captured to estimate abundance and density [40, 41]. This model assumes that animals move randomly and independently so its effectiveness varies by species [40].

Cameras can be deployed in different habitat types within an area to determine a species’ habitat use [2] and develop a habitat occupancy model. These models can be used to estimate occupancy of habitat by the species of interest, as well as its detectability within that habitat [42]. A selection of sites is sampled to estimate these values over a larger site or area. These models require repeated observation of the study sites [42]. Many cameras also have a time-lapse function, which allows them to be used to monitor a site over time [1]. Taking pictures at set time intervals can be useful for detecting changes at the site over time, whether those changes are animal activity, vegetation conditions, or weather events [1]. The field of camera trapping continues to evolve, and potential for the use and reuse of camera imagery datasets will only increase [2].

With this dataset, our goal was to determine whether the blunt-nosed leopard lizard was present in the study area of Elkhorn Plain. In addition, we wanted to determine if camera traps were an effective tool for detecting this particular species. The paired shrub-open design also allows habitat and microsite use to be examined with the dataset. Cameras successfully detected animal species in the open and were also effective under shrub canopies. With subsequent analyses, these data can be used to estimate microhabitat occupancy, and-to a lesser extent-spatial co-occurrence patterns. The animal/insect capture rate of 0.4 % suggests that extensive temporal and spatial sampling is required, particularly if the target animal species is relatively rare [5]. However, extended surveys of changes in populations and activity over time are possible because the camera traps can be deployed for a week or more with little to no maintenance [2, 37, 38]. Though this survey was only carried out between spring and summer, seasonal differences in animal abundances and densities may be assessed from the dataset. Additional cameras can also be easily deployed to extend the scale of the survey and sample a wider area [37, 38]. The inclusion of a date and time stamp on all of the pictures taken mean that time periods of specific activity for this animal species can also be described [43–45]. Limited, direct species behavior can also be described depending on how long animals spend within the frame of the camera-when animals remain in front of the camera for some time, cameras are triggered to take photographs frequently enough that animal activity and length of activity can be determined. Because this dataset provides information about the presences or absence of species at our study site, it may be useful as a starting point for researchers conducting more in depth surveys on the Elkhorn Plain. In addition, this dataset could be combined with other imagery datasets from the area to provide a more comprehensive picture of animal distribution and activity.

Image data can be useful in describing vegetation characteristics and short-term micro-environmental disturbances-we also propose that changes in vegetation over time may be described. Weather events such as rainfall and cloud patterns are recorded by these cameras. Temperature is encoded into every image, and these data can be extracted and used to evaluate ambient temperature differences between microenvironments and sites. Hence, image data from camera traps can be relevant to general ecology and vegetation science as well as conservation and wildlife biology.

Despite all their benefits, imagery datasets can be difficult to manage because of their sheer size. Manually processing hundreds of thousands of images is a time consuming process [8]. An automated approach, such as an automated algorithm, would greatly reduce the time and effort required for image processing. Imagery datasets such as this one would be useful as a training dataset for these algorithms as they are continually developed and improved.

### Availability of supporting data

The imagery dataset is deposited in the *GigaDB* repository [46], including the image, camera, and study design metadata. Images (in JPEG format) are organized into folders based on the specific deployment site and date. Basic information about the habitat at the camera microsite, the type of camera trap used at that microsite and its settings, and the location of the survey site are also provided.

### Ethics approval

Disturbance to plants and animals was kept to a minimum throughout the study. No plants or animals were collected during the course of the study. Authorization for this project was granted by Carrizo Plain National Monument, U.S. Bureau of Land Management, 3801 Pegasus Drive, Bakersfield, CA 93308–6837, USA.

## Abbreviations

JPEG: Joint photographic expert group
SD: Secure digital
